# Impact of an Andean breakfast on biochemistry and immunochemistry laboratory tests: an evaluation on behalf COLABIOCLI WG-PRE-LATAM

**DOI:** 10.11613/BM.2019.020702

**Published:** 2019-04-15

**Authors:** Wilson Bajaña, Eduardo Aranda, Maria Elena Arredondo, Lorena Michele Brennan-Bourdon, Marise Danielle Campelo, Edgar Espinoza, Silvia Flores, Patricia Ochoa, Veronica Vega, Beatriz Varela, Gabriel Lima-Oliveira

**Affiliations:** 1 International Laboratories Services, INTERLAB S.A., Guayaquil, Ecuador; 2 Department of Hematology-Oncology, School of Medicine, Pontificia Universidad Católica de Chile, Santiago, Chile; 3 BIONET S.A., Santiago, Chile; 4 Clinical Laboratory Network from the state of Jalisco, Public Health State Laboratory (LESP), Comisión para la Protección contra Riesgos Sanitarios del Estado de Jalisco (COPRISJAL), Mexico; 5 Clinical Laboratory Bioanalise, Teresina, Piaui, Brazil; 6 Servicio de Acreditación Ecuatoriano (SAE), Quito, Ecuador; 7 Universidad Peruana Cayetano Heredia, Lima, Perú; 8 Facultad de Medicina Universidad Católica de Cuenca, Cuenca, Ecuador; 9 Universidad de Guayaquil, Guayaquil, Ecuador; 10 LAC, Montevideo, Uruguay; 11 Section of Clinical Biochemistry; Department of Neurosciences, Biomedicine and Movement Sciences, University of Verona, Verona, Italy; 12 Latin American Working Group for Preanalytical Phase (WG-PRE-LATAM) of the Latin America Confederation of Clinical Biochemistry (COLABIOCLI), Montevideo, Uruguay

**Keywords:** blood specimen collection, diagnostic errors, fasting, postprandial period, reproducibility of results

## Abstract

**Introduction:**

In Andean countries, specifically in Ecuador, a food transition in the population has been observed because of economic growth. The Working Group for Preanalytical Phase in Latin America (WG-PRE-LATAM) of the Latin America Confederation of Clinical Biochemistry (COLABIOCLI) was established in 2017, and its main purpose is to study preanalytical variability and establish guidelines for preanalytical procedures in order to be implemented by clinical laboratories and healthcare professionals in Latin America. The aim of this study on behalf of COLABIOCLI WG-PRE-LATAM was to evaluate whether an Andean breakfast can interfere with routine biochemistry and immunochemistry laboratory tests.

**Materials and methods:**

We studied 20 healthy volunteers who consumed an Andean breakfast containing a standardized amount of carbohydrates, proteins and lipids. We collected blood specimens for laboratory tests before breakfast and 1, 2, and 4 hours thereafter. Significant differences between samples were assessed by the Wilcoxon ranked-pairs test.

**Results:**

The Andean breakfast statistically (P ≤ 0.05), modified the results of the following tests: triglycerides, insulin, cortisol, thyroid stimulating hormone, free thyroxine, total protein, albumin, urea, creatinine, lactate dehydrogenase, alkaline phosphatase, amylase, lipase, total bilirubin, direct bilirubin, iron, calcium, phosphorus, magnesium, and uric acid.

**Conclusions:**

Andean breakfast can influence the routine biochemistry and immunochemistry laboratory tests and might expose patient safety to some risks. Therefore, the COLABIOCLI WG-PRE-LATAM calls attention and highlights that the fasting time needs to be carefully considered when performing blood testing in order to prevent spurious results and thus, reduce laboratory errors.

## Introduction

The Andean countries are a group of South American nations that geographically comprise Venezuela, Colombia, Ecuador, Peru, Bolivia, northern Argentina and Chile. Agricultural production and livestock farming are the basis of the usual diet in these different countries. Nevertheless, improvement of the means of transport, transculturation due to the movements of the inhabitants, expanded distribution of agricultural products, food, and globalization, have contributed to generating a multicultural Andean diet.

Ecuador has a total area of 283,561 km^2^ (land + Galapagos Islands). Population is more than 16 million, with the majority living in the central provinces, in the Andes, and near the Pacific ocean coast (*1*). Economic growth has been the main cause of changes in the Latin American population dietary habits (*2*). Moreover, patients from Latin America have a particularly lifestyle that could promote changes in hormone levels, such as food intake, the ingestion of local tea infusions, sport practices, and also the altitude. The Andean breakfast in Ecuador include the *Bolones*, a fried green plantain dumplings typically stuffed with cheese or with *chicharrones*; briefly, *chicharrones* in Mexico and Central America are fried pork rinds, whereas, in Ecuador, these are actually chunks of deep fried fatty pork meat.

All the procedures preceding laboratory testing – the preanalytical phase – are responsible for the main source of laboratory variability (*3*, *4*). Fasting time for the majority of blood tests should be 12 h, whereas for lipid profile alone there is an exception based on European consensus (*5*, *6*). Presently, incorrect fasting status can be a source of errors that can jeopardize patient safety (*7*-*9*). The Latin American Working Group for Preanalytical Phase (WG-PRE-LATAM) of the Latin America Confederation of Clinical Biochemistry (COLABIOCLI), established in 2017, has the primary goal of studying preanalytical variability and establishing guidelines for preanalytical procedures to be applied by clinical laboratories and healthcare professionals in Latin America. This study on behalf of COLABIOCLI WG-PRE-LATAM was aimed at evaluating whether an Andean breakfast can interfere with routine biochemistry and immunochemistry laboratory tests done in either serum or plasma samples from the same individuals.

## Materials and Methods

### Study design

A total of 20 healthy volunteers (12 women and 8 men; average age was 32 (21-52) years) were selected from the personnel of the University of Guayaquil (Ecuador) and included in the study. Informed consent was obtained from all study subjects according to the 2013 Declaration of Helsinki and the protocol was approved by the Ethics Committee.

After a 12 hour overnight fast, the first blood sample was collected between 8:00 and 8:30 a.m. Then, immediately after the first venous blood collection, the subjects ate the Andean breakfast, containing standardized amounts of carbohydrates, protein, and lipids. Table 1 shows the exact composition of the Andean breakfast. Subsequent venous blood samples were performed at 1, 2, and 4 hours after breakfast.

**Table 1:** Nutritional composition of Andean breakfast

**Nutritional composition**	**Bolon chicharrón**	**Scrambled eggs**	**Yogurt**	**Apple juice**	**Total**
Number (overall weight, g)	1 (380)	1 (100)	1 (185)	1 (200)	4 (865)
Kcal	506	121	130	70.0	827
KJ	2117	505	545	293	3460
Protein (g)	6.8	12.1	6.0	0.0	24.9
Carbohydrate (g)	20.5	3.45	NA	17.0	41.0
Total lipids (g)	44.8	6.90	4.0	0.0	55.7
Cholesterol (mg)	42.2	448	10.0	0.0	500
NA – not available. Kcal - kilocalorie. KJ – kilojoule. Bolon chicharrón: a fried green plantain dumplings stuffed with *chicharrones*; briefly *chicharrones* in Ecuador are chunks of deep fried fatty pork meat.					

According to the international EFLM-COLABIOCLI recommendations, all venous blood sampling procedures were carried out by a single phlebotomist (*7*). In order to eliminate possible blood distribution interferences, all volunteers were kept in an upright sitting position for 15 min (*10*). Then, a vein was located on the forearm, however, in order to prevent venous stasis interference from the use of the tournique, and thus, avoid clench, a subcutaneous tissue transilluminator device (Venoscópio IV plus; Duan do Brasil, Brazil) was used (*11*, *12*). All blood samples were collected directly into one 3.5 mL evacuated tube containing gel separator and clot activator for serum samples, and into one 3.0 mL evacuated tube containing lithium heparin and gel separator for plasma samples (Vacumed®, FL Medical, Torreglia, Italy) using a 20 gauge needle in a closed evacuated system (FL Medical, Torreglia, Italy). To eliminate any possible interference due to either the contact phase or tissue factor, approximately 2 mL of blood were preliminarily collected in a discard tube without additive. The blood collection procedure was appropriately standardized in each phase, as already reported, particularly, in regard to sample processing, centrifugation and serum/plasma separation (*13*).

All samples were assayed in a single analytical run in the same instrument according to the manufacturer’s specifications and using proprietary reagents. The panel of tests that were performed and the instruments used by the International Laboratories Services Interlab S.A. (Guayaquil, Ecuador), an accredited laboratory due to International Organization for Standardization (ISO) 15189 standard, are shown in Table 2. The Ecuadorian Accreditation Service informed us that Ecuador has approximately 3900 clinical laboratories (3000 private and 900 public). However, the accreditation process according to ISO 15189 standard started in 2010, and presently, there are only eight accredited laboratories.

**Table 2:** Results of within-run precision by the internal quality control of the used instruments

**Instrument**	**Test**	**Method**	**IQC assigned values**	**CVa (%)**
**ARCHITECT C8000, ABBOTT**	CHOL	enzymatic, cholesterol oxidase / cholesterol esterase	4.82 mmol/L	0.5
	HDL	accelerator selective detergent, cholesterol oxidase / cholesterol esterase	0.80 mmol/L	1.7
	TG	enzymatic, glycerol phosphate oxidase	1.07 mmol/L	0.7
	TP	biuret	62.5 g/L	0.5
	Alb	bromocresol green, colorimetric	40.6 g/L	0.3
	Urea	UV, urease	5.42 mmol/L	1.5
	CREA	kinetic, alkaline picrate	232 µmol/L	0.9
	CRP	immunoturbidimetric	4.92 mg/L	0.7
	UA	enzymatic, uricase	0.33 mmol/L	0.6
	ALP	p-nitrophenyl phosphate	124 U/L	0.5
	AMY	CNPG3 substrate	76.7 U/L	1.0
	AST	IFCC, UV without P5P, 37 °C	34.1 U/L	1.3
	ALT	IFCC, UV without P5P, 37 °C	34.0 U/L	1.8
	GGT	L-Gamma-glutamyl-3-carboxy-4-nitroanilide substrate	55.0 U/L	1.1
	LD	IFCC, UV lactate-pyruvate	220 U/L	1.6
	LIP	quinone dye	43.0 U/L	0.8
	CK	N-acetyl-L-cysteine, NAC	137 U/L	1.3
	TBIL	diazonium salt	17.8 µmol/L	1.0
	DBIL	diazo reaction	18.6 µmol/L	1.1
	Phos	UV, phosphomolybdate	1.42 mmol/L	0.6
	Ca	arsenazo III, colorimetric	2.36 mmol/L	0.6
	Mg	arsenazo, colorimetric	0.91 mmol/L	1.3
	Fe	ferene, colorimetric	19.3 µmol/L	1.0
	Na	ion-selective electrode	143 mmol/L	0.2
	K	ion-selective electrode	4.00 mmol/L	0.2
	Cl	ion-selective electrode	99.0 mmol/L	0.2
**IMMULITE 2000 XP, Siemens**	TSH	chemiluminescence, biotin–streptavidin based	0.42 mIU/L	3.4
	FT4	chemiluminescence, biotin–streptavidin based	12.1 pmol/L	5.6
**Cobas e-601, Roche**	Ins	electrochemiluminescence, biotin–streptavidin based	176 mIU/L	1.6
	Cortisol	electrochemiluminescence, biotin–streptavidin based	94.4 nmol/L	1.7
IQC – internal quality control. CVa – analytical coefficient of variation. CHOL – cholesterol. HDL – high density lipoprotein. TG – triglycerides. TP – total protein. Alb – albumin. CREA – creatinine. CRP – C reactive protein. UA – uric acid. ALP – alkaline phosphatase. AMY – amylase. AST – aspartate aminotransferase. ALT – alanine aminotransferase. GGT – gamma glutamyl transferase. LD – lactate dehydrogenase. LIP – lipase. CK – creatine kinase. TBIL – total bilirubin. DBIL – direct bilirubin. Phos – phosphate. Ca – calcium. Mg – magnesium. Fe – iron. Na – sodium. K – potassium. Cl – chloride. TSH – thyroid stimulating hormone. FT4 – free thyroxin. Ins – insulin.				

The instruments were calibrated against appropriate proprietary reference standard materials and verified with independent third-party control materials from calibrator materials (Lyphochek^®^ Level 1 for routine biochemistry tests and Lyphochek Immunoassay Plus Control^®^, Level 1 for immunochemistry assays, Bio-Rad, California, USA), as recommended (*14*). The evaluation of the within-run precision by the internal quality control of the instruments used in this study, showed low coefficients of variation (Table 2).

### Statistical analysis

For assessing statistical difference between samples, the Wilcoxon ranked-pairs test was used in agreement with Simundic’s recommendations regarding sample size (*i.e.* less than 30), with a licensed statistical software (GraphPad Prism® version 5.01, La Jolla, CA, USA) (*15*). The level of statistical significance was set at P < 0.05. Mean % differences were determined according to the formula: mean % difference = [(× h after breakfast − basal) / × h after breakfast] × 100%.

Finally, the mean % differences from blood samples at 1, 2 and 4 hours after breakfast, were compared with the desirable specification for imprecision (DSI) derived from biologic variation (*16*). DSI was used as our criterion of acceptance in lipemia analytical interference testing, then interferograms were provided for each laboratory parameter with significant difference between basal and x h after Andean breakfast.

## Results

**Figure 1:**
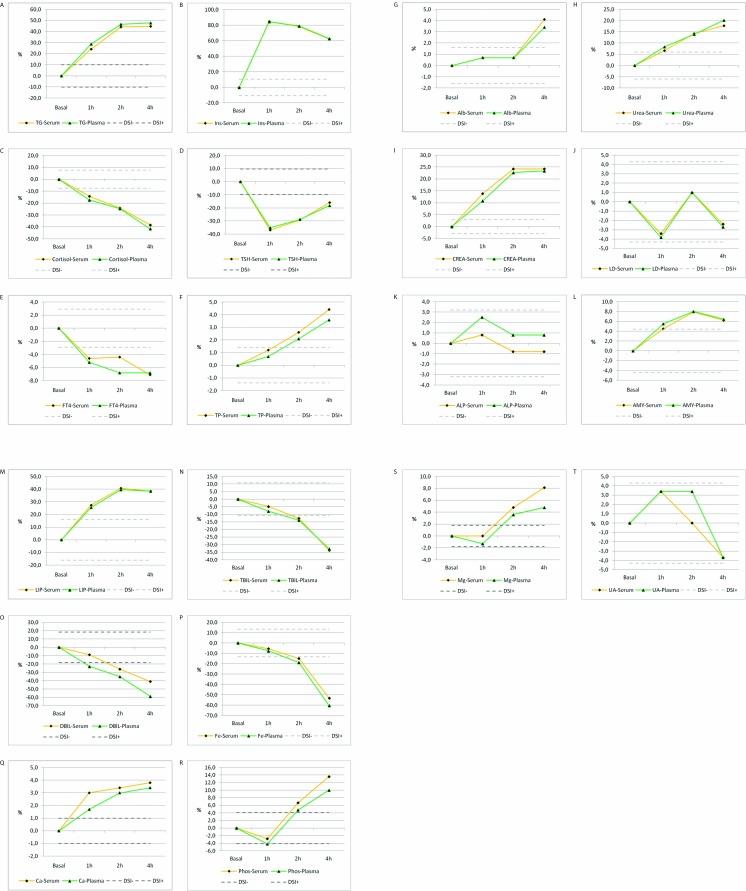
Interferograms. A: TG – triglycerides. B: Ins – insulin. C: cortisol. D: TSH – thyroid stimulating hormone. E: FT4 - free thyroxin. F: TP – total protein. G: Alb – albumin. H: Urea. I: CREA – creatinine. J: LD – lactate dehydrogenase. K: ALP – alkaline phosphatase. L: AMY – amylase. M: LIP – lipase. N: TBIL – total bilirubin. O: DBIL – direct bilirubin. P: Fe – iron. Q: Ca – calcium. R: Phos – phosphate. S: Mg – magnesium. T: UA – uric acid. Hours after the Andean breakfast (x-axis) are plotted against bias values (y-axis). Solid line – bias. Dashed lines - acceptable criteria based on desirable specification for imprecision (DSI) derived from biologic variation.

The results of the routine biochemistry laboratory tests are presented as median (interquartile range) in Table 3. Among all the results, statistical significant differences between basal and x h after the Andean breakfast were observed for the following parameters: triglycerides (TG), insulin (Ins), cortisol, thyroid stimulating hormone (TSH), free thyroxine (FT4), total protein (TP), albumin (Alb), urea, creatinine (CREA), lactate dehydrogenase (LD), alkaline phosphatase (ALP), amylase (AMY), lipase (LIP), total bilirubin (TBIL), direct bilirubin (DBIL), iron (Fe), calcium (Ca), phosphate (Phos), magnesium (Mg), and uric acid (UA) (Figure 1). In regards to serum *vs.* plasma (Table 3), both specimen types showed differences mainly related to the same tests, except for UA at 2 h (significant for serum, not for plasma). Moreover, plasma samples showed higher mean values for TP, clearly due to fibrinogen presence as expected. Mean values of serum ALP and AMY were slightly higher than plasma, whereas potassium (K) was significantly lower in plasma than in serum, an effect most probably due to lithium heparin an ion which competes with K for intracellular transport, and formation of coagulum, which is accompanied by extraction of potassium from platelets.

**Table 3:** Postprandial variation on laboratory tests after Andean breakfast

		**SERUM**				**PLASMA**			
	**Test** **(Unit)**	**BASAL**	**1h**	**2h**	**4h**	**BASAL**	**1h**	**2h**	**4h**
	**CHOL (mmol/L)**	5.0 (4.6 - 5.5)	5.0 (4.7 - 5.4)	5.0 (4.8 - 5.4)	5.0 (4.8 - 5.5)	5.0 (4.6 - 5.4)	4.9 (4.6 - 5.3)	5.0 (4.6 - 5.4)	4.9 (4.7 - 5.4)
**P**		-	0.777	0.667	0.720	-	0.856	0.738	0.113
	**HDL (mmol/L)**	1.2 (1.0 - 1.5)	1.2 (1.0 - 1.5)	1.2 (1.0 - 1.4)	1.2 (1.0 - 1.4)	1.2 (1.0 - 1.5)	1.2 (1.1 - 1.5)	1.2 (1.0 - 1.5)	1.2 (1.0 - 1.4)
**P**		-	0.897	0.737	0.528	-	0.764	0.629	0.472
	**TG** **(mmol/L)**	1.3 (1.1 - 1.7)	1.7 (1.4 - 2.2)	2.4 (1.5 - 3.1)	2.4 (1.4 - 3.2)	1.2 (1.0 - 1.6)	1.7 (1.3 - 2.1)	2.3 (1.8 - 3.2)	2.3 (1.3 - 3.1)
**P**		-	< 0.001	< 0.001	< 0.001	-	< 0.001	< 0.001	< 0.001
	**TP** **(g/L)**	72 (70 - 78)	73 (71 - 78)	74 (71 - 79)	76 (71 - 78)	75 (72 - 80)	75 (72 - 81)	76 (71 - 80)	78 (72 - 80)
**P**		-	0.033	0.002	0.008	-	0.034	0.005	0.002
	**Alb** **(g/L)**	45 (43 - 48)	45 (43 - 48)	45 (43 - 49)	47 (43 - 49)	44 (42 - 47)	45 (42 - 47)	45 (42 - 48)	46 (42 - 48)
**P**		-	0.513	0.081	0.033	-	0.647	0.760	0.021
	**Urea (mmol/L)**	3.7 (3.0 - 4.1)	3.9 (3.2 - 4.4)	4.3 (3.4 - 4.9)	4.5 (4.0 – 5.0)	3.5 (3.0 - 4.2)	3.9 (3.1 - 4.3)	4.1 (3.5 - 4.7)	4.4 (3.7 - 4.9)
**P**		-	0.001	0.001	< 0.001	-	0.009	< 0.001	< 0.001
	**CREA (µmol/L)**	66 (61 - 75)	77 (72 - 91)	88 (76 - 103)	88 (78 - 102)	66 (60 - 74)	74 (69 - 88)	86 (75 - 100)	87 (75 - 103)
**P**		-	< 0.001	< 0.001	< 0.001	-	< 0.001	< 0.001	< 0.001
	**CRP** **(mg/L)**	3.4 (1.0 - 6.2)	3.4 (0.9 - 6.1)	3.5 (0.9 - 6.0)	3.6 (0.9 - 6.1)	3.3 (0.9 - 6.0)	3.4 (0.9 - 5.9)	3.4 (0.8 - 5.9)	3.5 (0.7 - 5.9)
**P**		-	0.067	0.896	0.409	-	0.615	0.559	0.235
	**UA** **(µmol/L)**	280 (230 - 390)	290 (240 - 400)	280 (240 - 400)	270 (230 - 390)	280 (240 - 390)	290 (250 - 410)	290 (240 - 410)	270 (240 - 400)
**P**		-	0.003	0.987	0.007	-	0.005	0.003	0.005
	**ALP** **(U/L)**	73 (61 - 85)	74 (59 - 85)	72 (61 - 87)	72 (60 - 90)	69 (58 - 80)	71 (58 - 82)	70 (58 - 82)	70 (58 - 87)
**P**		-	0.030	0.003	0.001	-	0.007	0.003	0.001
		**SERUM**				**PLASMA**			
	**Test** **(Unit)**	**BASAL**	**1h**	**2h**	**4h**	**BASAL**	**1h**	**2h**	**4h**
	**AMY** **(U/L)**	62 (48 - 73)	65 (49 - 82)	67 (52 - 83)	66 (55 - 81)	61 (44 - 73)	64 (47 - 81)	66 (54 - 82)	65 (52 - 81)
**P**		-	< 0.001	< 0.001	< 0.001	-	< 0.001	< 0.001	< 0.001
	**AST** **(U/L)**	20 (17 - 27)	21 (17 - 27)	21 (17 - 28)	21 (17 - 26)	21 (17 - 27)	21 (17 - 28)	21 (16 - 28)	20 (16 - 25)
**P**		-	0.163	0.601	0.235	-	0.277	0.563	0.087
	**ALT** **(U/L)**	23 (17 - 38)	24 (17 - 39)	24 (17 - 39)	24 (18 - 38)	23 (17 - 38)	22 (17 - 38)	22 (18 - 38)	23 (18 - 38)
**P**		-	0.601	0.920	0.888	-	0.587	0.344	0.644
	**GGT** **(U/L)**	27 (15 - 41)	27 (15 - 41)	27 (17 - 41)	28 (15 - 42)	27 (15 - 41)	27 (14 - 39)	27 (15 - 39)	27 (15 - 41)
**P**		-	0.736	0.409	0.533	-	0.719	0.684	0.271
	**LD** **(U/L)**	176 (149 – 181)	171 (155 – 186)	178 (162 – 223)	172 (163 – 181)	178 (197 – 245)	172 (162 – 233)	180 (162 – 211)	173 (159 – 211)
**P**		-	0.033	0.011	0.043	-	0.028	0.012	0.039
	**LIP** **(U/L)**	19 (14 - 27)	26 (20 - 34)	32 (24 - 38)	31 (24 - 39)	19 (15 - 26)	25 (20 - 33)	31 (24 - 40)	31 (23 - 39)
**P**		-	< 0.001	< 0.001	< 0.001	-	< 0.001	< 0.001	< 0.001
	**CK** **(U/L)**	115 (62.9 – 156)	116 (65.9 – 157)	112 (63.5 – 155)	107 (61.8 – 149)	126 (65.9 - 151)	121 (70.0 – 153)	115 (65.3 – 151)	115 (66.5 – 151)
**P**		-	0.324	0.051	0.178	-	0.533	0.615	0.344
	**TBIL (µmol/L)**	10.8 (7.87 - 16.1)	10.3 (8.38 - 14.9)	9.58 (6.33 - 12.8)	7.70 (5.64 - 11.3)	10.9 (8.04 - 15.4)	10.1 (8.55 - 14.5)	9.58 (6.33 - 12.8)	8.21 (5.64 - 11.1)
**P**		-	0.248	0.001	<0.001	-	0.083	0.002	<0.001
	**DBIL (µmol/L)**	4.10 (3.08 - 5.47)	3.76 (3.08 - 4.96)	3.25 (2.39 - 4.28)	2.91 (2.22 - 3.42)	4.62 (3.25 - 5.47)	3.76 (3.08 - 5.13)	3.42 (2.39 - 4.45)	2.91 (2.22 - 3.76)
**P**		-	0.027	< 0.001	< 0.001	-	0.002	< 0.001	< 0.001
	**Phos (mmol/L)**	1.11 (1.07 - 1.17)	1.08 (1.01 - 1.16)	1.19 (1.02 - 1.27)	1.25 (1.13 - 1.36)	0.99 (0.96 - 1.07)	0.95 (0.87 - 1.03)	1.04 (0.90 - 1.12)	1.10 (1.01 - 1.21)
**P**		-	0.018	0.009	0.008	-	0.003	0.004	0.027
	**Ca** **(mmol/L)**	2.30 (2.26 - 2.41)	2.37 (2.31 - 2.48)	2.38 (2.33 - 2.49)	2.39 (2.32 - 2.45)	2.30 (2.24 - 2.38)	2.34 (2.28 - 2.45)	2.37 (2.30 - 2.46)	2.38 (2.31 - 2.44)
**P**		-	0.001	< 0.001	< 0.001	-	0.006	0.001	< 0.001
		**SERUM**				**PLASMA**			
	**Test** **(Unit)**	**BASAL**	**1h**	**2h**	**4h**	**BASAL**	**1h**	**2h**	**4h**
	**Mg** **(mmol/L)**	0.79 (0.75 - 0.83)	0.79 (0.77 - 0.82)	0.83 (0.81 - 0.86)	0.86 (0.82 - 0.87)	0.80 (0.76 - 0.84)	0.79 (0.78 - 0.81)	0.83 (0.81 - 0.85)	0.84 (0.81 - 0.87)
**P**		-	0.587	< 0.001	< 0.001	-	0.338	0.001	< 0.001
	**Fe** **(µmol/L)**	17 (13 - 19)	16 (12 - 19)	15 (11 - 18)	11 (9.0 - 15)	17 (12 - 19)	15 (12 - 18)	14 (11 - 18)	11 (9 - 15)
**P**		-	0.794	0.006	0.002	-	0.732	0.004	0.001
	**Na** **(mmol/L)**	138 (137 – 139)	139 (138 – 140)	139 (139 – 141)	139 (139 – 140)	138 (137 – 139)	139 (138 – 140)	139 (138 – 141)	139 (138 – 140)
**P**		-	0.764	0.762	0.865	-	0.694	0.703	0.865
	**K** **(mmol/L)**	4.08 (3.95 - 4.30)	4.13 (3.98 - 4.23)	4.20 (4.05 - 4.28)	4.18 (4.06 - 4.32)	3.73 (3.55 - 3.90)	3.65 (3.58 - 3.80)	3.69 (3.59 - 3.84)	3.71 (3.54 - 3.85)
**P**		-	0.904	0.586	0.184	-	0.313	0.520	0.384
	**Cl** **(mmol/L)**	104 (103 – 105)	104 (103 – 105)	104 (103 – 105)	104 (103 – 105)	104 (103 – 105)	104 (103 – 105)	104 (103 -105)	104 (103 – 105)
**P**		-	0.763	0.965	0.573	-	0.888	0.789	0.942
	**TSH (mIU/mL)**	1.82 (1.03 - 2.21)	1.33 (0.90 - 1.83)	1.41 (0.93 - 1.95)	1.57 (0.94 - 2.12)	1.89 (1.01 - 2.26)	1.40 (0.88 - 1.59)	1.47 (1.02 - 1.97)	1.60 (0.94 - 2.38)
**P**		-	< 0.001	0.005	0.031	-	<0.001	0.002	0.041
	**FT4 (pmol/L)**	13.6 (12.1 - 15.7)	13.0 (12.4 - 14.7)	12.9 (11.5 - 14.4)	12.7 (11.6 - 14.4)	14.2 (12.0 - 15.0)	13.5 (12.9 - 15.2)	13.3 (12.6 - 15.6)	13.3 (12.4 - 15.4)
**P**		-	0.011	0.009	0.014	-	0.016	0.034	0.036
	**Ins** **(mIU/L)**	11.4 (9.19 - 21.2)	75.5 (42.3 – 116)	52.1 (31.8 - 87.8)	30.2 (21.6 - 53.9)	11.7 (9.6 - 19.6)	75.0 (44.6 – 112)	55.0 (33.8 - 91.0)	31.4 (21.7 - 53.2)
**P**		-	< 0.001	< 0.001	< 0.001	-	< 0.001	< 0.001	< 0.001
	**Cortisol (nmol/L)**	242 (194 – 379)	212 (189 – 285)	195 (125 – 221)	175 (147 – 297)	242 (191 – 382)	206 (190 – 284)	194 (125 – 216)	171 (143 – 293)
**P**		-	0.017	0.010	0.009	-	0.016	0.001	0.013
Results are presented as median (interquartile range). P < 0.05 was considered statistically significant. 1h – 1 hour after breakfast. 2h – 2 hours after breakfast. 3h – 3 hours after breakfast. 4h – 4 hours after breakfast. CHOL – cholesterol. HDL – high density lipoprotein. TG – triglycerides. TP – total protein. Alb – albumin. CREA – creatinine. CRP – C reactive protein. UA – uric acid. ALP – alkaline phosphatase. AMY – amylase. AST – aspartate aminotransferase. ALT – alanine aminotransferase. GGT – gamma glutamyl transferase. LD – lactate dehydrogenase. LIP – lipase. CK – creatine kinase. TBIL – total bilirubin. DBIL – direct bilirubin. Phos – phosphate. Ca – calcium. Mg – magnesium. Fe – iron. Na – sodium. K – potassium. Cl – chloride. TSH – thyroid stimulating hormone. FT4 – free thyroxin. Ins – insulin.									

## Discussion

Our results mirror the metabolic course of TG, Ins and cortisol in the postprandial period (Figure 1A, 1B, and 1C), in accordance with previous findings that evidence the importance of the entero-endocrine system in nutrient sensing and assimilation (*17*). Moreover, according to Page *et al.*, “following a meal, the gastrointestinal hormones act in concert to regulate appetite, food intake, gastric acid secretion, gastrointestinal motility, and glucose homeostasis” (*17*). Each of the more than 20 known gastrointestinal hormones were initially thought to be produced by specific enteroendocrine cells, but it is now understood that these cells are flexible and express a range of peptide precursors (*18*). Moreover, since physicians presently request laboratory thyroid evaluation, avoiding fasting time, a comment is needed: our results (Figure 1D and 1E) showed a significant decrease of both TSH, and FT4 1h after breakfast and no return to baseline in the following 4 hours after food intake. The induced elevation of circulating somatostatin in the postprandial period and the consequent suppression of TSH could explain these results (*19*). This is in accordance with independent researchers that showed similar results by using different analytical methods for TSH and FT4 assays (*19*, *20*). Therefore, patients should be in a fasting condition to avoid both unclear thyroid laboratory results, and a misdiagnosis of hypothyroidism.

The Andean breakfast statistically modified both TP and Alb concentrations (Figure 1F and 1G). These results are in agreement with other studies, which showed that feeding stimulates Alb and other protein syntheses, since this event might improve the storage of essential amino acids (*21*-*24*). Moreover, Lima-Oliveira *et al.*, have shown a similar result for Alb after a light Italian meal, without significant changes in total protein (*13*). However, the Andean breakfast is richer in proteins than the light Italian meal – 24.9 g *vs.* 14.6 g – and this could explain the differences that were shown. Furthermore, the higher protein content of the Andean breakfast can also explain the urea and CREA results (Figure 1H and 1I). Thus, the outcome of such laboratory tests on non-fasting patients can interfere with the validity of the results and possibly, jeopardize patient safety.

Regarding enzymes, either transaminases (AST, ALT), gamma-glutamyl transferase or creatine kinase, did not show statistically relevant changes after the Andean breakfast (Table 3); LD and ALP showed statistical relevant changes after the Andean breakfast with variability in conformity with DSI (Table 3, Figure 1J and 1K). However, a significant increase in AMY and LIP activities was shown (Figure 1L and 1M). Boivin *et al.*, had experimentally demonstrated that the activity of pancreatic enzymes are influenced by diet type (*25*). This can explain why our results differ from Lima-Oliveira *et al.*, who did not evidence changes for either AMY or LIP in the Italian study with a light meal (*13*).

The significant decrease observed for bilirubin (Figure 1N and 1O) is in agreement with Meyer *et al*. (*26*). Iron fluctuations caused by intra-day variability and by the diet are thought to influence test results, and may affect clinical patient management (*27*). Moreover, the measurement of electrolytes is frequently requested and tested on patients avoiding fasting time. Our results showed a significant decrease of Fe concentration at 2h and 4h following the Andean breakfast (Figure 1P), whereas Ca, Phos and Mg significantly increased after food intake (Figure 1Q, 1R and 1S). Therefore, fasting should be required for evaluating bilirubin, iron, calcium, phosphate, and magnesium.

Differences demonstrated between serum and plasma are in agreement with the recent critical review published by Lima-Oliveira *et al*.: i) the concentration of proteins was higher in serum than in plasma; ii) differences between serum and plasma were observed for some enzymes tested; and iii) Ca, Phos and Mg were higher in serum than in plasma, since heparin can bind these ions (Figure 1) (*28*).

In conclusion, an Andean breakfast can affect routine biochemistry and immunochemistry laboratory tests and might jeopardize patient safety. Therefore, COLABIOCLI WG-PRE-LATAM calls attention and highlights that the fasting time needs to be carefully considered when performing tests, in order to prevent spurious results and reduce laboratory errors. Laboratory quality managers are encouraged to standardize the fasting requirements in their laboratory (*i.e.*, 12h) using the evidence reported above.
